# Towards Personalized Medicine for Chronic Liver Disease

**DOI:** 10.3390/jpm13101432

**Published:** 2023-09-25

**Authors:** Jingwen Gao, Chunfang Xu, Jinzhou Zhu

**Affiliations:** 1 Department of Gastroenterology, The First Affiliated Hospital of Soochow University, Suzhou 215000, China; 2 Suzhou Clinical Center of Digestive Diseases, Suzhou 215000, China

Chronic liver disease is a progressive deterioration of hepatic functions and a continuous process of inflammation, destruction, and regeneration of liver parenchyma, resulting in fibrosis and cirrhosis. The spectrum of etiologies encompasses metabolic disorders, viral infection, toxins, alcohol abuse, and genetic and autoimmune diseases.

In the past few decades, there has been significant progress in the field of genetics and artificial intelligence, which has provided new opportunities and methods for disease prevention, diagnosis, and treatment. Personalized clinical diagnosis and treatment have emerged as a crucial trend in the healthcare industry. Personalized medicine is a dynamic and rapidly developing approach in clinical practice that involves utilizing innovative technologies to make decisions in the screening, prevention, diagnosis, and treatment of disease. Personalized medicine, as the name suggests, involves developing unique medical plans for each patient based on their individual characteristics and health needs. Traditional medical models often rely on population statistics, while personalized medicine shifts the focus to the individual. By gaining a deep understanding of each patient’s genetic information, lifestyle habits, and environmental factors, medical practitioners can more accurately predict and diagnose diseases, develop more effective treatment plans, and improve treatment outcomes and patient satisfaction. The significance of personalized medicine for chronic liver disease lies not only in providing better outcomes for patients but also in avoiding unnecessary waste of medical resources. Additionally, personalized medicine helps prevent diseases from occurring by providing patients with earlier intervention and treatment opportunities, thereby reducing the risk and recurrence rate of diseases.

In the recent Special Issue titled “Towards Personalized Medicine for Chronic Liver Disease” in the *Journal of Personalized Medicine*, six research articles focused on clinical epidemiology and machine learning in the field of chronic liver disease were published.

The hepatic venous occlusion type of Budd–Chiari syndrome and pyrrolizidine alkaloid-induced hepatic sinusoidal obstructive syndrome share similar clinical features and imaging findings, leading to misdiagnoses. In a six-central case-control study, Tong et al. [1] compared the clinical manifestations, laboratory tests, and imaging of patients with two syndromes. They discovered that, in addition to exposure to pyrrolizidine alkaloid-containing plants, local hepatic vein stenosis and the presence of collateral circulation of hepatic veins are the most significant imaging evidence to identify two syndromes.

Wedge hepatic vein pressure is used to evaluate the portal pressure in patients with chronic sinusoidal portal hypertension. Owing to hepatic sinusoidal obstruction syndrome, it is uncertain if this tool was feasible in patients with acute portal hypertension. Cheng et al. [2] aimed to assess the agreement between wedge hepatic vein pressure and portal pressure in patients with hepatic sinusoidal obstruction syndrome and with decompensated cirrhosis. They found that wedge hepatic vein pressure in patients with pyrrolidine alkaloid-induced hepatic sinusoidal obstruction syndrome did not reflect portal pressure as accurately as in patients with virus- or alcohol-related cirrhosis, primarily due to the overestimation of portal pressure.

Portal vein thrombosis is a prevalent complication in cirrhotic patients and exacerbates portal hypertension, thereby leading to a range of severe complications. Ding et al. [3] aimed to construct a logistic-regression-based model to predict portal vein thrombosis in cirrhotic patients. They collected clinical data from 656 cirrhotic patients with or without portal vein thrombosis from two hospitals. They identified serum albumin, D-dimer, portal vein diameter, splenectomy, and esophageal and gastric varices as key variables. Based on the clinical and imaging findings, the nomogram might serve as a prediction model for portal vein thrombosis in cirrhotic patients.

Previous studies have demonstrated that clinical models based on machine learning outperformed models based on traditional logistic regression. However, there have been no previous reports on automated machine learning and cirrhosis. Therefore, Yu et al. [4] conducted this hospital-based case-control study to develop automated machine learning models for predicting 30-day mortality in patients with non-cholestatic cirrhosis. In the study, the prediction model based on the XGBoost algorithm exhibited superior performance compared to existing scoring systems for predicting 30-day mortality in patients with non-cholestatic cirrhosis. It also shows the potential of automated machine learning in future medical applications.

Even though nonalcoholic fatty liver disease has been commonly associated with obesity, it can also affect individuals with a lean body composition. Previous studies concerned with predictive models for nonalcoholic fatty liver disease in lean populations are rare. Thus, Liu et al. [5] collected data from a single-central cross-sectional study involving 5037 lean individuals to develop a nomogram for predicting the risk of nonalcoholic fatty liver disease in lean subjects. The nomogram was developed based on seven predictors: alanine aminotransferase, total cholesterol, triglycerides, low-density lipoprotein cholesterol, creatinine, uric acid, and hemoglobin A1C. The authors believe that this model may aid in identifying lean nonalcoholic fatty liver disease during future population-based screening.

The advancement of new applications in ultrasound imaging in recent years promotes the role of ultrasound in managing various pathologies, particularly in chronic liver disease. As a novel elastography technique, shear wave dispersion is an emerging imaging technology that enables the evaluation of the dispersion slope of shear waves. Analyzing the dispersion characteristics of shear waves can indirectly provide information about tissue viscosity, thus offering biomechanical insights into hepatic pathology, e.g., necroinflammation. In the Special Issue, Garcovich et al. [6] reviewed the feasibility of liver viscosity based on the preliminary findings of both animal and human studies.

Although personalized medicine has tremendous potential in theory, it faces some challenges in practical application. Firstly, personalized medicine incurs higher costs, including expenses related to genetic sequencing and bioinformatics analysis, which limits its widespread adoption on a large scale. Secondly, personalized medicine requires the establishment of large-scale genomic databases and bioinformatics databases to support doctors’ decision-making and predictions. Moreover, personalized medicine also involves ethical and privacy protection issues, necessitating the establishment of relevant laws, regulations, and management systems.

However, with the continuous advancement of technology and cost reduction, the prospects of personalized medicine remain vast. More and more medical institutions and research organizations are investing in the research and application of personalized medicine, driving the development and innovation of related technologies. In the future, personalized medicine is expected to become the mainstream model in the field of healthcare, providing patients with better health management and treatment services.

## Data Availability

Not applicable.
